# Levels of PAHs in the Waters, Sediments, and Shrimps of Estero de Urias, an Estuary in Mexico, and Their Toxicological Effects

**DOI:** 10.1100/2012/687034

**Published:** 2012-09-10

**Authors:** Foday M. Jaward, Henry A. Alegria, Jose G. Galindo Reyes, Armando Hoare

**Affiliations:** ^1^Department of Environmental and Occupational Health, College of Public Health, University of South Florida, 13201 Bruce B. Downs Boulevard, MDC 56, Tampa, FL 33612, USA; ^2^Department of Environmental Science, Policy & Geography, University of South Florida St. Petersburg, 140 7th Avenue South, St. Petersburg, FL 33701, USA; ^3^Facultad de Ciencias del Mar, Universidad Autónoma de Sinaloa, Paseo Claussen s/n, 82000 Mazatlán, SIN, Mexico

## Abstract

PAHs were measured in water, sediment, and shrimps of Estero de Urias, an estuary in Sinaloa, Mexico, during the rainy and dry seasons, and analyzed for eleven PAHs routinely detected in samples. Phenanthrene was the most dominant congener in the water, sediment, and shrimp samples comprising about 38, 24, and 25%, respectively, of the eleven PAHs detected, followed by pyrene and naphthalene in water and sediment samples, and pyrene and fluorine in the shrimp samples. Total PAH concentrations ranged from 9 to 347 ng/L in water, 27 to 418 ng/g in sediments, and 36 to 498 ng/g in shrimps. The sources of contamination are closely related to human activities such as domestic and industrial discharge, automobile exhausts, and street runoff. High concentrations were also measured during the rainy season and during the first quarter of the year. Toxicity tests were also carried out, exposing fish embryos and juvenile shrimps to some of these PAHs. Fish embryos exposed to PAHs showed exogastrulation, while juvenile shrimps showed significantly lower growth rates than controls. DNA and protein alterations were also observed. These toxicity tests indicate that PAH concentrations measured could be dangerous to some aquatic organisms, particularly during early stages of development.

## 1. Introduction

Polycyclic aromatic hydrocarbons (PAHs) comprise a class of pollutants that persist in the environment and pose risks to animals, plants, and people at elevated concentrations [1]. They are byproducts of incomplete combustion of natural or anthropogenic sources whose environmental behavior has been investigated for more than 40 years. Because of their persistence, toxicity, carcinogenicity, and mutagenicity, they continue to attract the interest of researchers [2, 3]. Natural sources are mainly due to pyrolytic processes such as forest fires and volcanic activities. Anthropogenic sources include motor vehicles, domestic burning of coal and wood for space heating, power generation via combustion of coal and oil, incineration, wood burning, cooking, smoking, and burning of natural gas [1, 4, 5]. In industrial countries, especially in urban areas, anthropogenic sources are by far the greatest contributors to environmental burden. The highest ambient levels occur in winter months (consistent with increased combustion-derived PAH emissions in colder periods) and tend to be concentrated in urban areas, which can in turn influence PAH concentrations in rural areas [6]. Residues of these pollutants enter coastal ecosystems via continental runoff, atmospheric deposition, municipal and industrial effluents, and often by direct discharge. Use of fossil fuels like fuel-oil (which is an important source of PAHs) has been restricted and even forbidden in developed countries. However, elsewhere including Mexico, their use is not properly regulated or supervised. 

PAHs have been detected in sediments, water, and crustaceans as mixtures and typically cooccur with other contaminants. However, recent studies have reported diverse kinds of pollution in this estuary, such as pesticides and polychlorinated biphenyls (PCBs) [7, 8] and associated toxic effects on aquatic organisms [9], but to the knowledge of the authors there have been no reports on the levels and effects of PAHs in different matrices. This study provides a comprehensive dataset for the first time on levels of PAHs analyzed in this region. Water, sediment and shrimp samples were taken from Estero de Urias, an estuary in Sinaloa, Mexico, once a month in February, May, September, and December 2007 at several stations. The samples were collected during the rainy and dry seasons and analyzed for eleven PAHs (naphthalene, fluorene, phenanthrene, anthracene, fluoranthene, pyrene, chrysene, benzo[b]fluoranthene, benzo[a]pyrene, benzo[ghi]perylene, and Indeno[1,2,3-c,d]pyrene) routinely detected in samples. Toxicity tests were also carried out, exposing fish embryos and juvenile shrimps to three of these PAHs. 

## 2. Materials and Methods

### 2.1. Chemicals and Reagents

All solvents used in this work were of analytical grade and purchased through VWR Scientific (NY, USA). Silica (60 mesh), alumina, and sodium sulfate manufactured by Baker (NJ, USA) were purchased through VWR Scientific (NY, USA). Deuterated PAH cocktail standard (ES-2044) containing acenaphthylene-*d8*, pyrene-*d10*, phenanthrene-*d10*, naphthalene-*d8*, fluoranthene-*d10*, benzo[a]pyrene-*d12*, and benzo[*ghi*]perylene-*d12* was used as the internal standard mix and was obtained from Cambridge Isotope Laboratories (CIL, Andover, MA, USA). The surrogates (acenaphthene-*d10*, anthracene-*d10*, fluorene-*d10*, perylene-*d12*, and chrysene-*d12*) were also purchased from CIL. RNA-ase and proteinase-K were purchased from Sigma-Aldrich Co. USA.

### 2.2. Study Site and Sampling

**Figure 1:**
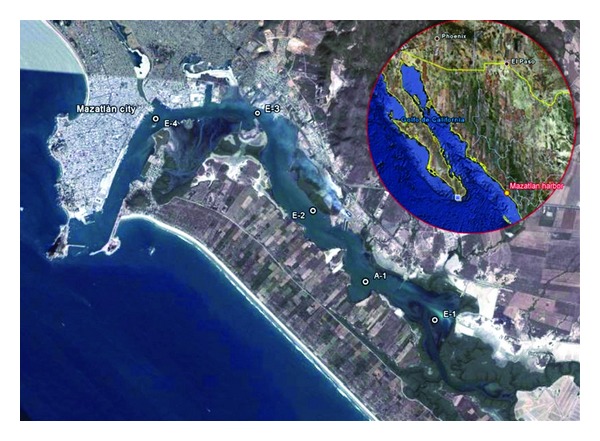
Estero de Urias estuary in Sinaloa, Mexico.

The study was conducted in the Estero de Urias estuary. This estuary is located at the entrance of the Gulf of California, behind the city of Mazatlan (Figure 1). The harbor and several industries, including a power plant of Comision Federal de Electricidad (the federal electricity company), facilities of PEMEX (the Mexican state oil company), and tuna and shrimp packing factories, frequently discharge their waste into this water body. Therefore, Estero de Urias has been receiving diverse pollutants for many years. Even so, it is a place for harvesting aquatic species of economic value, like shrimp and several fishes. In order to determine the spatial and seasonal variability of pollution by PAHs, water, sediment, and shrimp samples were taken once a month in February, May, September, and December 2007 at several stations (Figure 1) using Niskin bottles, a Van veen grab, and a hand fishing net for water, sediments, and shrimps, respectively [7]. Shrimp samples were only collected from station A1, located close to an artisanal fishing area. Samples were transported to the laboratory and stored at −20°C until analyses within 48 hours. 

### 2.3. Sample Extraction and Analysis

In the laboratory, the samples were processed by several methods to extract and analyze PAHs. Extraction and cleanup procedures have been described in detail elsewhere [7]. Briefly, for water samples, pollutants were extracted by acetone:hexane using a liquid-liquid separation system, whereas for sediments and shrimps, samples were first freeze-dried, then soxhlet-extracted with n-hexane. Prior to extraction, each sample and blank was spiked with a range of deuterated PAH compounds (acenaphthene-*d10*, anthracene-*d10*, fluorene-*d10*, chrysene-*d12*, and perylene-*d12*) to monitor analytical recovery. The extracts were reduced in volume on a rotary evaporator, solvent exchanged to hexane, and interfering compounds removed by column chromatography using 10 g silica and 5 g alumina (and 0.5 cm anhydrous Na_2_SO_4_ at the top of the column to prevent the column from contact with air) and eluting the compounds of interest with 100 mL 1 : 1 mixture of hexane : DCM. The eluent was blown down using a TurboVap II concentration workstation (Hopkinton, MA, USA) with a gentle stream of nitrogen, transferred to 4 mL vials and blown down under a gentle stream of nitrogen. They were further cleaned by gel permeation chromatography using 6 g of Biobeads SX 3 (BioRad, Hertfordshire, UK). Sulfur compounds in sediment samples, which interfere with later analysis, were removed by soaking the extracts with activated copper powder. The final volume was made up in 100 *μ*L of isooctane using a Turbovap II concentration workstation with a gentle stream of nitrogen and spiked with a range of deuterated PAHs (acenaphthylene-d8, pyrene-d10, phenanthrene-d10, naphthalene-d8, fluoranthene-d10, benzo[a]pyrene-d12, and benzo[ghi]perylene-d12) as internal standards. The sample extracts were analyzed using an Agilent GC 7890A using splitless injection on a 30 m HP5-ms column (0.25 mm i.d., 0.25 *μ*m film thickness) and helium as carrier gas. The oven was programmed at 60°C for 2 min, ramped at 20°C min^−1^ to 180°C, and further ramped at 6°C min^−1^ to 280°C and held for 20 min. This was coupled to an Agilent 5975C mass selective detector operated in electron impact (EI) mode using selected ion monitoring (SIM). The injector temperature was set at 290°C and the interface temperature at 280°C. Identification and quantification were carried out against 5 calibration standards of known concentrations using the internal standard method. Eleven PAHs (naphthalene, fluorene, phenanthrene, anthracene, fluoranthene, pyrene, chrysene, benzo[b]fluoranthene, benzo[a]pyrene, benzo[ghi]perylene and indeno[1,2,3-c,d]pyrene) were analyzed using a calibration curve for each PAH congener and all samples were quantified for these PAHs using the calibration curves. 

**Figure 2:**
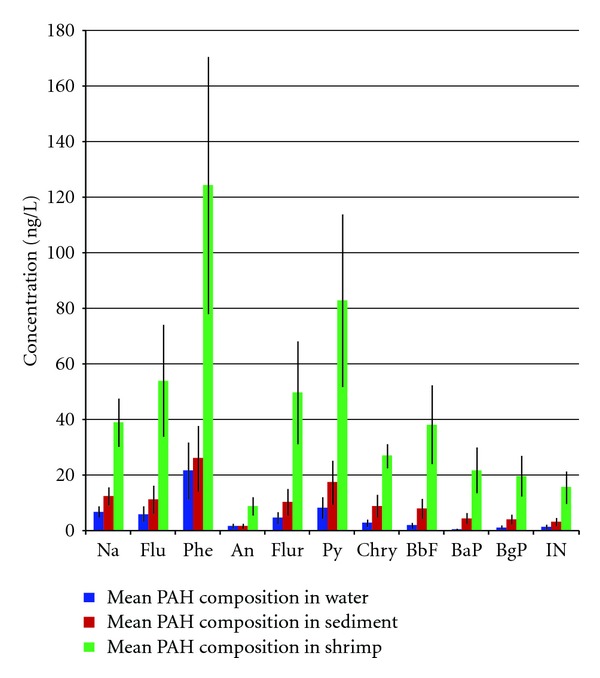
Mean PAH concentrations with 95% confidence interval.

**Figure 3:**
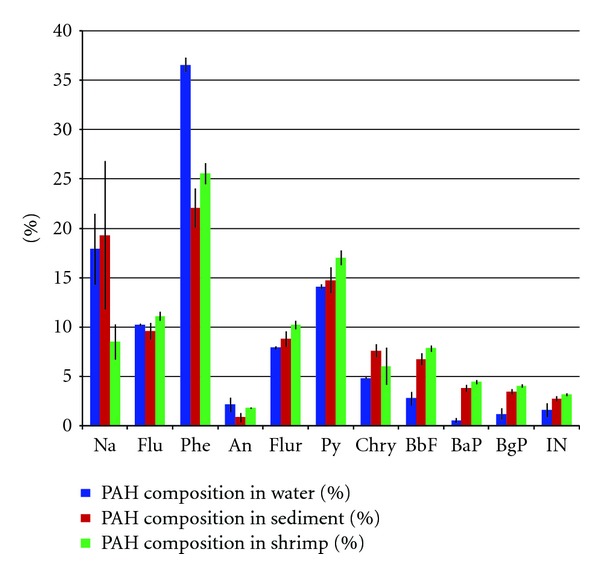
Mean percent PAH composition with 95% confidence interval.

PAH concentrations in sediment and shrimp were calculated by dividing the amounts in extracts by the actual weight of sediment or shrimp tissue extracted after adjusting for moisture (on a dry weight) while levels in water were calculated by dividing amount in extracts by volume of water extracted. A total of 11 PAH congeners regularly detected in samples were quantified. These are naphthalene, fluorene, phenanthrene, anthracene, fluoranthene, pyrene, chrysene, benzo[b]fluoranthene, benzo[a]pyrene, benzo[ghi]perylene, and Indeno[1,2,3-c,d]pyrene. In the discussion that follows, Σ11PAH refers to the sum of the 11 congeners measured in this study. The congener specific and mean PAH concentrations in the samples are given in Table 1 and Figure 2. The compositional profiles in all matrices are given in Figure 3. In all three matrices, phenanthrene was by far the most dominant congener (Figures 2 and 3).

### 2.4. QA/QC

For every 5 samples, an analytical blank was processed comprising of a laboratory or a field blank. The blanks were treated the same way actual samples were treated. A peak was positively identified if it was within ±0.05 min of the retention time in the calibration standard and quantified only if the S/N ≥ 3, and the ratio of the target ion to its qualifier ion was within ±20% of the standard value. The PAHs present in the appropriate blanks were subtracted from those in the sample extracts. The method detection limits (MDLs) were calculated as the mean blank + 3 × SD. The method detection limits for all PAHs analyzed ranged from 0.4–3 ng/L in water and 0.4 to 3 ng/g in sediment and shrimp. Overall recovery was determined by spiking each sample and blank with 100 ng of PAH recovery standards (anthracene-d10, fluorene-d10, chrysene-d12, and perylene-d12) before extraction. The average recovery was 102% (range 68–112%) for all recovery standards.

### 2.5. Toxicity Tests

Several laboratory experiments were performed to determine toxic effects of PAHs found on morphological development of fish embryos (*Cupleidae spp*.) and growth rate, amount of DNA and proteins of white shrimp (*Litopenaeus vannamei*). In the first experiment, fish embryos were exposed (incubated) for 24 hours to discrete amounts of water and sediment extracts from Estero de Urias. All experiments were done in triplicates and the mean values reported. As a control treatment, filtered sea water (FSW) was used, and dimethyl sulfoxide (the solvent used to dissolve the extracts) was added to a final concentration of 0.1%. Fish embryos were exposed and controls were incubated at 25°C for 24 hours until the control embryos reached the last gastrula stage. After this period, the embryos (control and exposed) were fixed with 1% glutaraldehyde solution in FSW. The fixed embryos were observed under an Olympus CK2 inverted microscope to identify developmental abnormalities such as vegetalization (development of higher proportion of endodermic structures) and exogastrulation (evagination of archenteron). 

In a second study, the effects of PAHs on growth rate and protein level were investigated by exposing juvenile shrimp (*L. vannamei*) to naphthalene, phenanthrene, and pyrene (detected in 85% of samples and in high concentrations at Estero de Urias), and creosote for 21 days. The effects of PAHs on DNA amount were also investigated. Five shrimps each were distributed in 20 L aquaria. Each aquarium was dosed with 2 mL acetone solutions of naphthalene, phenanthrene, and pyrene to final concentrations of 0.125 and 0.250 *μ*g/L of phenanthrene and naphthalene, respectively, and 225 and 450 *μ*g/L of pyrene. All experiments were done in triplicates and the mean values were reported. The chemicals were obtained from Cambridge Isotope Laboratories (CIL, Andover, MA, USA). The control was shrimp in FSW aquaria with 3 mL of acetone. After the period of exposure, the shrimps were sacrificed to quantify the growth rate (weight increase per day) and protein amount from abdominal muscle by Folin-Ciocalteu method [10]. Also, DNA from the shrimp pleopods (swimming legs) was extracted, following the phenol-chloroform method proposed by Davis et al. [11]. The extracts were purified with RNA-ase and Proteinase-K, both from Sigma-Aldrich Co., to eliminate RNA and protein residues, and the DNA quantity was determined by High Performance Liquid Chromatography (HPLC), using a Shimadzu LC10A HPLC apparatus coupled to a Shimadzu RF-551 fluorescence detector. 

## 3. Results and Discussions

### 3.1. PAH Concentrations and Compositional Profile

In water, PAH concentrations in water range from 9 to 347 ng/L (Table 1). These levels compare well with studies by Zhu et al. in the Zhejiang Province in China [12] but are higher than data reported by Kanchanamayoon and Tatrahun [13]. Lowest values were generally measured in May and September at all stations. For all PAHs, the highest concentrations correspond to the end of the rainy season. The rainy season in the study region spans between September/October and January/February. With storms and hurricanes in October–November, the high values in February and December could be attributed to surface runoff. Another plausible reason for the high levels is due to the high ship traffic (fossil fuels being source of PAHs) in the early months of the year. The PAH profile was dominated by the low molecular weight congeners with order of importance as follows: phenanthrene, 38%; pyrene, 14.5%; naphthalene, 12%; fluorene, 10.5%; and fluoranthene, 8%. The high molecular weight congeners were very low or below detection limit. This is expected because of the relative high affinity of the heavier congeners to sorb to particulate matter due to their high partition coefficients [14]. 

PAH concentrations in sediment samples range from 27 to 418 ng/g. Phenanthrene was by far the most dominant congener (418 ng/g) followed by pyrene (279 ng/g) and naphthalene (201 ng/g) (Table 1). These levels compare well with studies by Zhu et al. [12], but are higher than the study by Moriwaki et al. [15]. Though the levels of the high molecular weight compounds are low in water, they are much higher in the sediment samples compared to the water samples. The PAH profile was dominated by phenanthrene, 24%; pyrene, 16%; naphthalene, 11.5%; fluorene, 10.5%; and fluoranthene, 9.6%. The sources of contamination are closely related to human activities such as domestic and industrial discharge, automobile exhausts, and street runoff. Lowest values were measured in September followed by December at all stations, while high values were generally measured in February and May at all stations.

Shrimp samples were only collected from station A1, which is closer to an artisanal fishing area. The eleven PAH compounds analyzed were all detected in shrimp samples with concentrations ranging from 36 to 498 ng/g (Table 1). Phenanthrene, 26%; pyrene, 17%; fluorene, 11%; fluoranthene, 10%; and naphthalene, 8% were the most abundant, ranging from 200 to 498 ng/g (Table 1). Again, even though the levels of the high molecular weight compounds are low in water, they are much higher in the shrimp samples. 

### 3.2. Spatial and Seasonal Distribution

Spatially, the highest PAH concentrations were recorded at sites E2 and E3, which are close to the electric power plant which burns fuel oil, for both sediment and water samples (see Table 1). It could be that pyrolysis is the principal pollution source of PAHs to this estuary. Other studies have reported that pyrolysis of fossil fuels is the principal source of hydrocarbons in sediments of aquatic ecosystems. The PAHs determined in this work are among organic pollutants that are moderately persistent and hard to be degraded by fungi and bacteria. For example, the half-life of phenanthrene is 17 weeks [16, 17]. The degradation rate of PAHs is determined by many environmental factors and physical-chemical proprieties of each compound, but among the more important are temperature, oxidation-reduction potential, molecular size, and ring number. Estero de Urias has a wide range of temperature (from 18°C in February to 33°C in September). 

Spatial and seasonal statistical analyses of the total PAHs in both the water and sediment samples were separately carried out. The two-factor ANOVA model II without replication by Zar [18] followed by the pair-wise comparison Tukey test when applicable was used. For the total PAHs in the water, there was statistical significance at the seasonal factor level (*F*_3,9_ = 19.52; *P* = 0.003) but not at the spatial factor level (*F*_3,9_ = 1.32; *P* = 0.3277). Further analysis showed that total PAH means in the water showed statistical significance at a significance level of 5% between the months: February and December versus May and September. For ΣPAHs in the sediment, there was also statistical significance at the seasonal factor level (*F*_3,9_ = 24.09; *P* = 0.001) but not at the spatial factor level (*F*_3,9_ = 1.73; *P* = 0.2304). February and May ΣPAHs values test to be similar with each other but different than ΣPAHs values for December and September, which test to be similar with each other at a significance level of 5%.

From the results obtained from field samples, Estero de Urias is moderately polluted by PAHs, and levels detected are comparable to those reported for other estuaries [19]. The results also indicate that the levels of these PAHs are higher in the rainy months than the dry months. This is likely due to increased input via runoff from sources around the estuary. Levels were also higher in locations closer to industrial and municipal drainages (samples E2 and E3), again indicating increased input via surface runoff. Very high levels of individual PAHs have been reported in various studies globally. For example, Woodhead and Matthiessen reported concentrations of individual PAHs ranging from 100 to 1000 ng/g (22,100 ng/g for pyrene) in estuaries in England [20], whereas for a number of sites in USA, such as Long Island Sound, Salem Harbor, and Boston Harbor, total PAH concentrations were in excess of 10,000 ng/g [21]. Another contribution to high PAH concentrations found in Estero de Urias during February could be likely due to pollution by ship. There is high ship traffic in the Mazatlan harbor during the first quarter of the year. However, Rust et al. and Varanasi et al. [22, 23] have reported low degradation rates of these compounds by metabolic activity of bacteria, polychaete, and other benthic organisms because for each 10°C decrease in temperature, metabolism decreases by about two to three times. In Estero de Urias, water temperature in February was around 18°C, whereas in September it was about 33°C. 

### 3.3. Toxicity Tests

#### 3.3.1. Exposure of Fish Embryos to Water and Sediment Extracts from Estero de Urias

**Figure 4:**
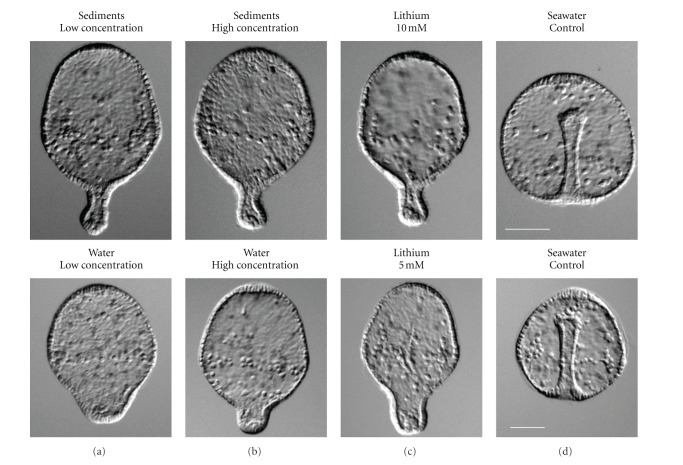
Deformities and abnormalities (exogastrulation) observed in fish embryos exposed to water and sediment extracts from Estero de Urias.

The results of the experiment with fish embryos are shown in Figure 4. Some deformities (exogastrulation) occurred in embryos exposed to extracts of water and sediments from Estero de Urias. These indicate that some abnormal process occurred during the development of the embryo similar to when they are exposed to lithium chloride (LiCl), a well-known vegetalizing agent [24]. The frequency of deformed embryos ranged between 10 to 25%. The lowest value was observed in embryos exposed to water extracts from September samples (low PAH concentration) and the highest from sediment extracts in February (high PAH concentration).

The toxicity test performed with fish embryos indicates that concentrations found in this work could be dangerous to some aquatic organisms, particularly during early stages of development such as embryo, larvae, and so forth. The presence of PAHs can be a potential risk because some PAHs have been reported as carcinogens (in both aquatic organisms and humans), and some are classified as mutagenic [25, 26]. Another risk factor is the ability of benthic organisms to metabolize these compounds. Some aquatic invertebrates, for example, certain polychaetes, are able to degrade PAHs [27, 28], but many others are not able to do this well, for example, bivalve mollusks [29]. PAHs are metabolized by phase 1 enzymes of cytochrome P450 mixed function oxygenase system, and excreted as hydroxylated metabolites [30]. However, the cytochrome P450 system, while metabolizing PAHs to more easily excretable compounds, produces a range of carcinogenic and mutagenic intermediates such as diols and epoxides that can lead to induction of carcinomas via formation of PAH-DNA adducts [31, 32]. Other studies report that even low PAH concentrations in sediments (2-3 ng/g) are capable of inducing cytochrome P450 enzymes in fishes [33]. There are also well-established associations between neoplastic and preneoplastic diseases in flatfish livers, cytochrome P450 induction and areas of PAHs contaminated sediments [34, 35]. However, PAH concentrations in sediments that have been associated with liver cancer in fishes and other aquatic organisms are variable due to the simultaneous presence of other pollutants, such as polychlorinated biphenyls (PCBs) and some pesticides that also induce P450. This situation complicates the picture.

### 3.4. Growth Rate, Protein Level, and DNA Amount in Shrimp Exposed to PAHs

**Figure 5:**
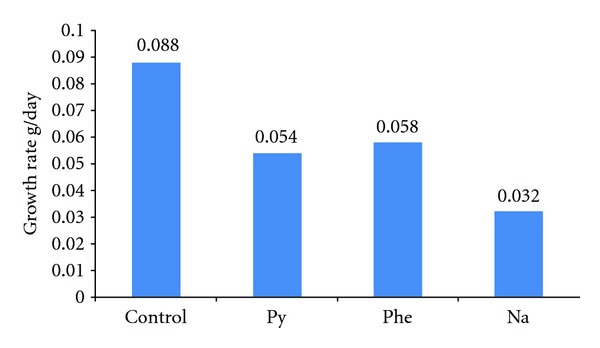
Mean growth rate of *L. vannamei* exposed to PAHs for 21 days.

**Figure 6:**
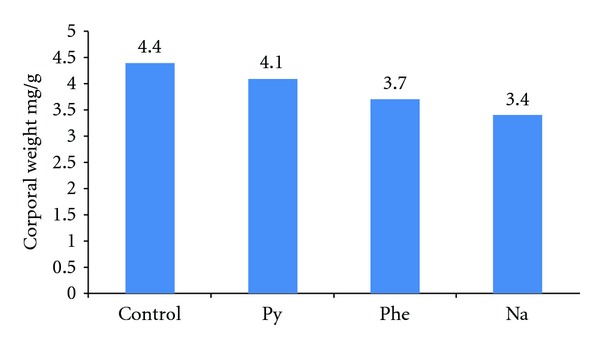
Mean protein concentration of *L. vannamei* exposed to PAHs for 21 days.

**Figure 7:**
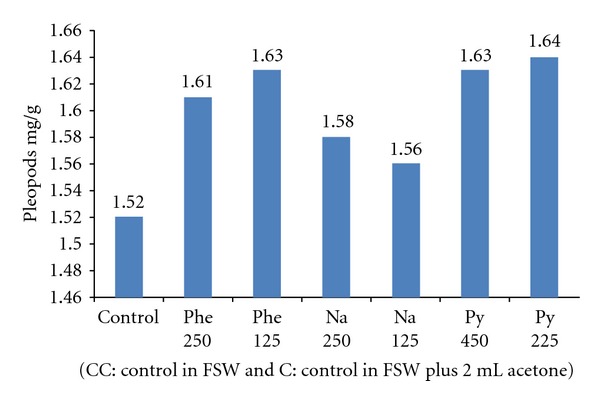
Mean DNA concentration of *L. vannamei* exposed to phenanthrene, naphthalene, and pyrene.

**Figure 8:**
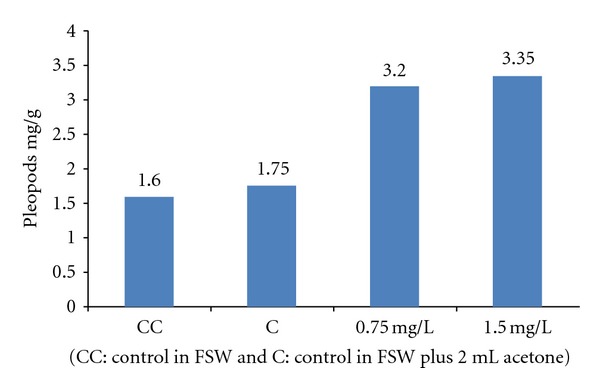
Mean DNA concentration of *L. vannamei* exposed to creosote for 21 days.

The results of the experiment to quantify the growth rate and protein level of shrimp exposed to PAHs are shown in Figures 5 and 6 where protein level and growth rate were lower in shrimps exposed to PAHs than controls. The DNA amount from shrimps exposed to diverse concentrations of phenanthrene, naphthalene, pyrene, and creosote are shown in Figures 7 and 8. In all cases, mean DNA amounts from experimental shrimps were higher than controls. These tests with pure PAHs lead us to conclude that at least PAHs are partially responsible for the observed effects. 

Concerning DNA damage of shrimp exposed to PAHs, results suggest that these pollutants also can be potential carcinogenic substances, because the DNA level of shrimp exposed was significantly higher than controls, which indicates possible DNA damage and start of carcinogenic processes. However, others have reported that many factors are involved in carcinogenic development in aquatic organisms exposed to toxic substances, such as development stage, nutritional and health condition and also the pollutant concentration and length of exposition [24].

## 4. Conclusions

The results indicate that the levels of these PAHs are higher in the rainy months than the dry months, which is likely due to increased input via runoff from sources around the estuary. Levels were also higher in locations closer to industrial and municipal drainages pointing to increased input via surface runoff. Another contribution to high PAH concentrations found in Estero de Urias could be likely due to pollution by ship. There is high ship traffic in the Mazatlan harbor during the first quarter of the year. Toxicity test performed with fish embryos indicates that concentrations found here could be dangerous to some aquatic organisms, particularly during early stages of development such as embryo and larvae. The presence of PAHs can be a potential risk since some have been reported as carcinogens (in both aquatic organisms and humans) and some are classified as mutagenic. Damage of DNA in shrimps exposed to PAHs suggests that these pollutants can be potential carcinogenic substances. The pollution load of PAHs in this estuary and others along the Gulf of California must therefore be reduced to improve the health of coastal ecosystems, thereby guaranteeing safe human consumption of sea foods as far as PAHs are concerned.

## Figures and Tables

**Table 1:** Congener specific PAH concentrations in water, sediments, and shrimps.

PAH concentrations in water samples (ng/L)											
Station/month	Na	Flu	Phe	An	Flur	Py	Chry	BbF	BaP	BgP	IN
E1-Feb	8.4	5.9	21.1	1.8	4.6	8.1	2.8	2.0	0.6	1.4	1.6
E1-May	3.4	1.3	4.6	<dl	1.0	1.8	0.6	<dl	<dl	<dl	<dl
E1-Sep	3.5	1.5	5.4	0.5	1.2	2.1	0.7	0.5	<dl	<dl	<dl
E1-Dec	8.9	7.4	26.6	2.3	5.8	10.2	3.5	2.5	0.7	1.7	2.0

E2-Feb	13.7	18.9	68.0	5.8	14.8	26.2	9.1	6.4	1.8	4.4	5.2
E2-May	4.2	1.5	5.2	<dl	1.1	2.0	0.7	0.5	<dl	<dl	<dl
E2-Sep	3.3	1.3	4.6	<dl	1.0	1.8	0.6	<dl	<dl	<dl	<dl
E2-Dec	10.3	9.3	33.4	2.8	7.3	12.9	4.5	3.1	0.9	2.2	2.5

E3-Feb	12.5	16.1	57.9	4.9	12.6	22.3	7.7	5.4	1.5	3.7	4.4
E3-May	3.7	1.7	5.9	0.5	1.3	2.3	0.8	0.6	<dl	<dl	0.5
E3-Sep	3.5	1.6	5.6	0.5	1.2	2.2	0.7	0.5	<dl	<dl	<dl
E3-Dec	8.8	7.0	25.2	2.1	5.5	9.7	3.4	2.4	0.7	1.6	1.9

E4-Feb	12.2	13.5	48.5	4.1	10.5	18.6	6.5	4.5	1.3	3.1	3.7
E4-May	3.4	1.4	4.9	<dl	1.1	1.9	0.7	0.5	<dl	<dl	<dl
E4-Sep	3.4	1.3	4.8	<dl	1.0	1.8	0.6	<dl	<dl	<dl	<dl
E4-Dec	8.0	7.0	25.2	2.1	5.5	9.7	3.4	2.4	0.7	1.6	1.9

Total PAH	111.1	96.3	346.8	27.4	75.4	133.4	46.2	31.1	9.0	19.7	23.6

PAH concentrations in sediment samples (ng/g)											

E1-Feb	18.2	17.2	39.5	2.8	15.8	26.4	13.6	12.2	6.9	6.3	5.0
E1-May	8.4	9.2	21.1	1.5	8.4	14.0	7.3	6.5	3.7	3.3	2.6
E1-Sep	3.5	2.0	4.6	<dl	1.8	3.1	1.6	1.4	0.8	0.7	0.6
E1-Dec	22.3	2.4	5.4	<dl	2.2	3.6	1.9	1.7	0.9	0.9	0.7

E2-Feb	19.3	29.6	68.0	4.9	27.2	45.4	23.5	20.9	11.9	10.8	8.6
E2-May	13.7	19.9	45.7	3.3	18.3	30.4	15.7	14.1	8.0	7.2	5.7
E2-Sep	3.3	2.3	5.2	<dl	2.1	3.5	1.8	1.6	0.9	0.8	0.7
E2-Dec	14.3	2.6	5.9	<dl	2.4	4.0	2.1	1.8	1.0	0.9	0.7

E3-Feb	22.8	26.2	60.2	4.3	24.1	40.1	20.7	18.5	10.6	9.5	7.6
E3-May	12.5	25.2	57.9	4.2	23.1	38.6	19.9	17.8	10.1	9.2	7.3
E3-Sep	3.5	2.1	4.9	<dl	2.0	3.3	1.7	1.5	0.9	0.8	0.6
E3-Dec	13.9	2.4	5.6	<dl	2.2	3.7	1.9	1.7	1.0	0.9	0.7

E4-Feb	17.5	15.9	36.5	2.6	14.6	24.3	12.6	11.2	6.4	5.8	4.6
E4-May	12.2	21.1	48.5	3.5	19.4	32.3	16.7	14.9	8.5	7.7	6.1
E4-Sep	3.4	2.0	4.6	<dl	1.8	3.1	1.6	1.4	0.8	0.7	0.6
E4-Dec	12.1	2.1	4.8	<dl	1.9	3.2	1.7	1.5	0.8	0.8	0.6

Total PAH	201.0	181.9	418.4	27.1	167.4	278.9	144.3	128.7	73.4	66.4	52.6

PAH concentrations in shrimp samples (ng/g)											

A1-Feb	38.1	59.7	137.4	9.9	54.9	91.6	26.6	42.3	24.1	21.8	17.3
A1-May	51.3	77.6	178.4	12.8	71.4	119.0	33.4	54.9	31.3	28.3	22.4
A1-Sep	30.2	28.1	64.5	4.6	25.8	43.0	24.1	19.9	11.3	10.2	8.1
A1-Dec	37.1	51.0	117.4	8.4	47.0	78.3	24.2	36.1	20.6	18.6	14.8

Total PAH	156.8	216.4	497.8	35.8	199.1	331.8	108.3	153.2	87.3	79.0	62.6

Na: naphthalene; Flu: fluorine; Phe: phenanthrene; An: anthracene; Flur: fluoranthene; Py: pyrene; Chry: chrysene; BbF: benzo[b]fluoranthene; BaP: benzo[a]pyrene; BgP: benzo[ghi]perylene; IN: Indeno[1,2,3-c,d]pyrene.
